# Can the Concentration of Citric Acid Affect Its Cytotoxicity and Antimicrobial Activity?

**DOI:** 10.3390/dj10080148

**Published:** 2022-08-09

**Authors:** Luciano Giardino, Luigi Generali, Paolo Savadori, Mirela Cesar Barros, Leticia Lobo de Melo Simas, Jolanta Pytko-Polończyk, Wojciech Wilkoński, Vasudev Ballal, Flaviana Bombarda de Andrade

**Affiliations:** 1 Independent Researcher, 88900 Crotone, Italy; 2 Department of Surgery, Medicine, Dentistry and Morphological Sciences with Transplant Surgery, Oncology and Regenerative Medicine Relevance (CHIMOMO), University of Modena and Reggio Emilia, 41124 Modena, Italy; 3 Department of Biomedical, Surgical, and Dental Sciences, Universita degli Studi di Milano, 20133 Milano, Italy; 4 Department of Operative Dentistry, Endodontics and Dental Materials, Bauru School of Dentistry, University of São Paulo, Bauru 17012-901, Brazil; 5 Department of Integrated Dentistry, Dental Institute, Faculty of Medicine, Jagiellonian University Medical College, 31-008 Cracow, Poland; 6 Independent Researcher, 34-100 Wadowice, Poland; 7 Department of Conservative Dentistry & Endodontics, Manipal College of Dental Sciences, Manipal Academy of Higher Education, Manipal 576104, India

**Keywords:** citric acid, confocal laser scanning microscopy, cytotoxicity, dentinal tubules, minimal inhibitory concentration, minimal bactericidal concentration

## Abstract

Background: There has been no unanimity concerning the ideal concentration of citric acid for safe use in clinical practice. This study evaluated the cytotoxicity and the antibacterial activity in infected dentinal tubules of 10% and 1% citric acid (CA) solutions. Methods: The cytotoxicity of CA solutions in DMEM (diluted 1/10, 1/100) was assessed in L-929 fibroblasts. A broth macrodilution method (MIC and MBC) was used to assess CA antibacterial concentration. The antimicrobial activity of CA solutions was also evaluated after their final rinse inside root canals in previously *Enterococcus faecalis*-contaminated dentinal tubules. Ten infected dentine samples were rinsed for 5 min with 5% NaOCl and subsequently with 1% citric acid for 3 min. Another 10 were rinsed with 5% NaOCl and 10% citric acid for 3 min; the remaining four specimens were utilized as positive controls. Two uncontaminated specimens were used as negative controls. After LIVE/DEAD BacLight staining, the samples were assessed using CLSM to analyze the percentage of residual live and dead cells. Results: Both undiluted and diluted CA solutions showed severe toxicity; no changes from normal morphology were displayed when diluted 1/100. The MIC and MBC of CA were 6.25 mg/mL and 12.50 mg/mL, respectively. CA solutions demonstrated significantly low levels of bacterial counts than the positive control group, reporting a value of 9.3% for the 10% solution versus the 1% solution (35.2%). Conclusions: Despite its valuable antimicrobial properties, the cytotoxic effects of citric acid should be considered during endodontic treatment.

## 1. Introduction

The key objective of root canal treatment is to eliminate/minimize the bacteria within the infected root canal system to enhance the healing of periapical tissues by providing a three-dimensional obturation [1]. However, researchers studying the remaining bacteria and the degree of cleaning the root canal, irrespective of the instrumentation technique implemented, have demonstrated inadequate cleaning and ineffective disinfection of the root canal system [2]. Owing to these shortcomings, recent research on the quality and efficacy of root canal irrigation has concentrated on irrigants with improved cleaning and antibacterial action as an imperative complement to mechanical cleaning and shaping. Sodium hypochlorite (NaOCl) and ethylenediaminetetraacetic acid (EDTA) are the gold standards for dissolution of necrotic tissue, eliminating the smear layer, and destroying the microbial colonies in the complex root canal system [3]. Although EDTA has been widely proposed for a final rinse of mechanically instrumented root canals [4], its shortcoming lies in slight/no antimicrobial effectiveness [5] and insubstantial smear layer removal in poorly penetrable parts of the root canal due to its increased surface tension [6]. EDTA, when used in combination with rotary instruments during shaping procedures, possesses a lubricant action when it comes in contact with the instrument, thus reducing stress generated by the rotary instruments without affecting their cyclic fatigue resistance, improving cleansing of the root canal system, as demonstrated by Zanza et al. [7]. For these reasons, it is still widely used clinically regardless of its shortcomings. Owing to the findings of the previous literature, 10% citric acid (CA) has been suggested as an adequate substitute to EDTA to better eliminate the smear layer from the root canal walls [8,9,10]. The reason is probably due to CA acidic pH, which increases the elimination of inorganic components, namely, calcium [11].

The lower ability of the smear layer elimination of EDTA compared to citric acid could be explained because EDTA’s properties are self-restricting due to its neutral pH, according to Jaiswal et al. [12]. Hennequin et al. [13] demonstrated that the effectiveness of the citric acid solution did not increase with concentration because the demineralizing effect was similar for CA at 50% pH 0.8 and 20% pH 1.3, with both of these being less effective if compared to CA 30% pH 1.1. Haznedaroğlu [14] later tested different citric acid solutions and confirmed this data using original and buffered pH solutions. That investigation found that citric acid with its original pH was as efficacious as higher concentrations in removing the peripheral/surface smear layer [14]. Furthermore, some researchers observed more erosive effects on dentin for 5% CA and 10% CA than 1% CA [15]. As a result, a lower concentration of citric acid (1%) has been proposed as a valuable alternative for the removal of the smear layer from dentin walls clinically [16], eluding erosion of root canal dentin [17].

Moreover, citric acid solution demonstrated antibacterial activity on anaerobic microorganisms [18,19] and oral biofilm [20], which is elucidated by its low pH values that enhance extracellular acidulation [21], altering the membrane permeability of bacteria and creating an environment unfavorable for their growth. Therefore, a helpful root canal irrigant should possess antibacterial properties, solvent effects on organic and inorganic tissues, and no cytotoxic effects on the host cells. Some authors [22,23] have studied the toxicity of citric acid in the past years, demonstrating high toxicity across multiple cell lines at the suggested concentrations. To date, however, the limited and not recent literature on this topic and a non-uniform survey methodology employed with conflicting results do not help resolve the doubts on its safe use in clinical practice. According to Peters [24], biocompatibility is a pivotal aspect to be cautiously assessed prior to releasing a chemical into the market. Then, the purpose of the current study was to assess the cytotoxicity and antibacterial activity of a less concentrated CA solution, comparing it to a more concentrated CA irrigating solution. The null hypothesis tested was that both concentrations of CA have similar antimicrobial activity.

## 2. Materials and Methods

The cytotoxicity of serial dilutions of 10% citric acid solution (201-069-1, Sigma-Aldrich, St. Louis, MO, USA) was measured in vitro according to an established method [25]. The mouse fibroblasts BS CL 56 (http://www.ibvr.org/Resources/CELLCULTURES_CATALOG.pdf, accessed on 6 July 2022), obtained from Istituto Zooprofilattico Sperimentale della Lombardia e dell’ Emilia Romagna (I.Z.S.L.E.R., Brescia, Italy), was grown at 37 °C in minimum essential medium (MEM) (41090036, Thermo Fisher Scientific, Rodano, Italy), enhanced with 10% fetal bovine serum (26140079, Gibco, Life Technologies Srl, San Giuliano Milanese, Italy), 100 IU/mL penicillin (J63901.14, Thermo Fisher Scientific, Rodano, Italy), 100 µg/mL streptomycin (11860038, Gibco, Life Technologies Srl, San Giuliano Milanese, Italy), and 0.25 µg/mL amphotericin B (15290026, Gibco, Life Technologies Srl, San Giuliano Milanese, Italy) in a 5% CO_2_ environment with 95% humidity for 24 h [26]. Cell cultures were developed into a near-confluent monolayer in culture dishes (20035, SPL Life Sciences Co. Ltd., Naechon-Myeon, Pocheon-si, Gyeonggi-do, Korea). Three dishes for each specimen were prepared. In addition, three dishes were used for the negative and positive controls. The medium was then removed and restored with a fresh MEM culture medium consisting of 0.5 mL of the undiluted 10% solution and 0.5 mL of its dilutions 1/10 (0.05 mL) and 1/100 (0.005 mL). Additionally, negative (cells and culture medium alone) and positive control (cells and latex gloves) groups were added. All experimental procedures were carried out in triplicate for test samples and controls [25].

Cytotoxic effects were evaluated microscopically by a qualitative method [25] (page 9). Cells cultured in the presence of various formulations of liquid were scrutinized at 200× magnification under an inverted optical microscope (OPTIKA XDS-3 Pomerania (BG), Italy) and evaluated for general morphology, vacuolation, detachment, cell lysis, and membrane integrity after the 24 and 48 h incubation periods [24]. The shift from normal morphology of the negative control was rated on a reactivity grade from 0 to 4. Degrees of toxicity were scored as illustrated in Table 1.

### 2.1. Assessment of Antimicrobial Activity

#### Macrodiluition Test

The minimal inhibitory concentration (MIC) and the minimal bactericidal concentration (MBC) of the chelator under examination were derived by the broth macrodilution method as per the guidelines of the Clinical and Laboratory Standards Institute (https://clsi.org/media/1928/m07ed11_sample.pdf, accessed on 9 February 2022). Screw cap tubes consisting of 2 mL of brain heart infusion (BHI) broth (50-488-527, Difco, Detroit, MI, USA) were utilized, and precise volumes and concentrations of CA were added to the tubes, generating a twofold serial dilution. The bacterial strain *Enterococcus faecalis* (*E. faecalis*) ATCC 29212 (American Type Culture Collection) was reactivated. Tubes were incubated at 37 °C, and successive 24 h cultures were executed to derive maximum bacterial growth. The bacterial culture was diluted to a standard 4 McFarland (12 × 10^8^ CFU/mL) and further diluted to a 5 × 10^5^ CFU/mL concentration in each tube containing chelators and broth. As some chelators modified the medium, it was essential to analogize the turbidity readings in the spectrophotometer (Bel Photonics do Brasil Ltd.a, Osasco, Brazil, nº SF325NM) at 540 nm before and after the incubation to ascertain the tubes with bacterial growth. Tube readings determined the minimum inhibitory concentration MIC for citric acid. The time required to promote growth was 24 h at 37 °C. Negative (no inoculum) and positive bacterial growth (inoculum without the antimicrobial) controls were carried out. After reading the final absorbances, the MBC, defined as the lowest concentration of an antimicrobial substance able to kill 99.9% of the initial inoculum, was determined by subculturing 50 µL of microbial suspension from tubes onto brain heart infusion (BHI) agar plates (DF0418-15-9, Difco, Detroit, MI, USA). The agar plates were incubated at 37 °C for 24 h to establish the MBC of citric acid over *E. faecalis*.

### 2.2. Sample Selection for Intratubular Decontamination

The current study was authorized by the Ethics Committee of the University of São Paulo (USP), Brazil (CAAE: 39330620.0.0000.5417). The numbers of specimens were calculated before the experiment, using G * Power v 3.1 for Mac software (Heinrich Heine, University of Düsseldorf, Germany), picking the Wilcoxon–Mann–Whitney test from Student’s *t*-test. The alpha-type error of 0.05, the beta power of 0.95, and the N2/N1 ratio of 1 were also specified. The test showed a total of 10 specimens for each group as the ideal size to observe significant differentiation. Preliminary tests were carried out on top of the sample estimation. Twenty-six single-rooted human teeth were chosen after radiographic examination in buccolingual projection from a stock of extracted teeth and kept in a 0.1% thymol solution (201-944-8, Sigma-Aldrich, St. Louis, MO, USA) at 5 °C for up to 15 days. The teeth had matured apices and single canals, with no signs of root resorption or calcifications in the root canals.

### 2.3. Sample Preparation and Infection

The teeth were decoronated, and the apical thirds of the roots were separated using a double-sided diamond disc 0.10 × 22 mm (KG Sorensen, Cotia, SP, Brazil) connected to a low-speed handpiece under water coolant to normalize root lengths to 8 mm. The root canals (i.e., the inside diameter of the dentin cylinders) were prepared with a Gates Glidden drill size 4 (Dentsply Maillefer, Ballaigues, Switzerland) to homogenize a diameter of 1.1 mm [27,28]. The smear layer was eliminated using an ultrasonic bath with 5% NaOCl (425044, Sigma-Aldrich, St. Louis, MO, USA), 17% EDTA (Fórmula e Ação, São Paulo, SP, Brazil), and physiological saline solution for 5 min apiece. Afterward, the samples were coated with red nail polish (Colorama, Rio de Janeiro, RJ, Brazil) to avoid external bacterial contamination and limit intratubular bacterial penetration to the main root canal. The specimens were sterilized separately in microtubes (Axygen Scientific, Union City, CA, USA) with distilled water in an autoclave (Cristofoli, Campo Mourão, PR, Brazil) at 121 °C for 24 min, incorporated in a sterilized brain heart infusion (BHI) culture medium (50-488-527, Difco, Detroit, MI, USA), and submitted to an ultrasonic bath for 10 min to ensure maximum infiltration of the culture broth into the dentinal tubules [28]. Confocal laser scanning microscopy (CLSM) analysis carried out arbitrarily in two specimens, used as negative controls (C−), established their sterility. The bacterial strain *E. faecalis* ATCC 29212 (American Type Culture Collection) was reactivated. The culture was modified to the #1 McFarland scale (3 × 10^8^ CFU/mL) with the help of a spectrophotometer (Bel Photonics do Brasil Ltd.a, Osasco, SP, Brazil, nº SF325NM) and incubated at 37 °C for seven hours to attain exponential bacterial growth. The contamination of samples lasted for five days at 37 °C, as per to the centrifugation protocol elicited by Andrade et al. [28] and Ma et al. [29]. Next, the samples were retrieved from the microtubes and prepared for treatment with the irrigants on the fifth day. The experiments were carried out under aseptic conditions inside a laminar flow hood.

### 2.4. Antimicrobial Test

For the irrigation of each group, the specimens were positioned in a sterilized metallic device inside a laminar flow. The apical third of each sample was sealed with a composite layer (Opticore classic, IDS, Savona, Italy) to mimic an ex vivo closed-end model [30]. They were randomly distributed among two experimental groups (n = 10). Four more samples not subjected to irrigation treatment were considered positive controls (C+). Every specimen of the experimental groups was rinsed with a 30-gauge side-vented needle (Ultradent Products Inc., South Jordan, UT, USA) throughout the root canal length. In group I (NaOCl + citric acid 10%), each sample was subjected to irrigation using 5 mL of 5% NaOCl (425044, Sigma-Aldrich, St. Louis, MO, USA) for 5 min, followed by 5 mL of citric acid 10% (Farmácia Specífica, Bauru, SP, Brazil) for 3 min. In group II (NaOCl + citric acid 1%), each sample was subjected to irrigation using 5 mL of 5% NaOCl (425044, Sigma-Aldrich, St. Louis, MO, USA) for 5 min, followed by 5 mL of citric acid 1% (Farmácia Specífica, Bauru, SP, Brazil) for 3 min. In both groups, after using 5% NaOCl, this solution was eliminated from the root canal of each specimen by adding 5% sodium thiosulfate (10102-17-7, Sigma-Aldrich, St. Louis, MO, USA) for 5 min [5].

### 2.5. Antimicrobial Evaluation of the Solutions by CLSM

After exposure to different irrigants, the specimens were split lengthwise in an Isomet machine (Buehler, IL, USA). Next, every sample was exposed to 17% EDTA for 5 min and flushed to sterile saline to eliminate the smear layer produced during cutting [31]. The samples were then subjected to staining for 20 min in a dark environment with 30 μL of the LIVE/DEAD^™^ BacLight bacterial viability kit (Invitrogen Molecular Probes, Eugene, OR, USA). This kit consists of SYTO 9^™^ Green Stain to stain the viable bacteria and Propidium Iodide Red Stain^™^ for the dead bacteria. Finally, the specimens were positioned on a glass slide with immersion oil and perceived using a Leica TCS-SPE confocal microscope (Leica Microsystems GmbH, Mannheim, Germany) employing 40× oil lenses at a step size of 1 μm and 1024 × 1024 pixel format.

Eight sequential images were procured from the specimens: four images of each side of the root canal, two in the cervical portion and two in the apical part; and four images of each area, two in a superficial area and two in a deeper area relative to the main root canal, totaling 80 images from each group and 32 from positive controls. The images were procured and fragmented using the Leica Application Suite-Advanced Fluorescence (LAS AF, Leica Microsystems GmbH, Mannheim, Germany) software and exported to TIFF format [28]. These images were then exported to the bioImage_L v21 software to assess the percentage of viable (green-stained) and non-viable (red-stained) bacteria [32]. The percentage of bacterial viability determined for the eight images (cervical, apical, superficial, and deep) of each specimen was computed using the following formula:

$$percent=\frac{volume\;of\;green\;\left[\mu m^{3}\right]}{volume\;of\;red+green\;\left[\mu m^{3}\right]}\;\times \;100$$

### 2.6. Statistical Analysis

The normality of data distribution was performed using the Shapiro–Wilk test. For intratubular infection, descriptive statistics were expressed, reporting the median and 95% confidence intervals (CI). Comparison among groups was carried out using the Mann–Whitney test for independent samples. As previously reported, the Wilcoxon signed-rank test was used for intra-group comparisons, comparing the percentage of viable bacteria among different root areas and between surface and deep layers [30]. Statistical analysis was performed using the GraphPad Prism 5.0 software (GraphPad San Diego, CA, USA). The level of significance was established at *p* = 0.05.

## 3. Results

### 3.1. Cytotoxicity

**Figure 1:**
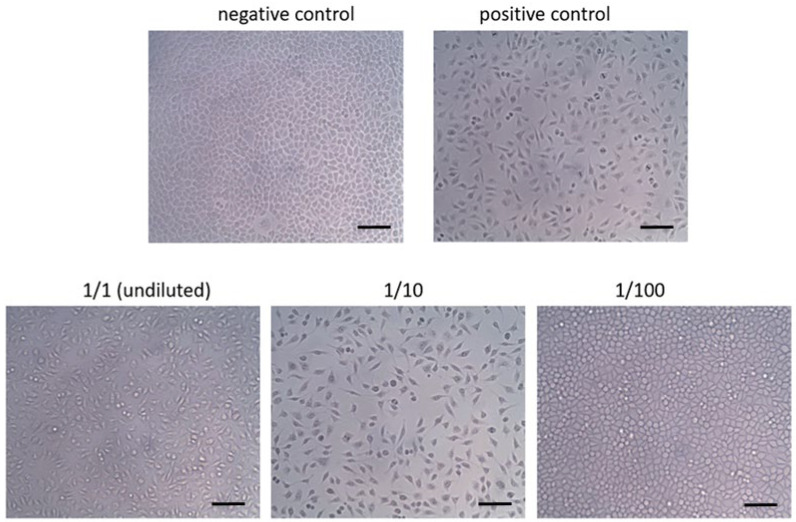
Cells treated with the tested solutions and controls (200×). Bars: 40.0 µm.

As shown in Figure 1, cells exposed to fresh medium (negative control) demonstrated healthy growth all through the study (grade 0). Conversely, those exposed to latex gloves (positive control) showed a severe toxic response (grade 4).

The cells treated with undiluted and diluted 1/10 test solution showed severe changes (grade 4) from the normal morphology of the negative control. Inversely, the cells treated with the 1/100 dilution did not display any changes from normal morphology (grade 0), similarly to the negative control.

### 3.2. Antimicrobial Analysis

The MIC and MBC of citric acid acquired by the macrodilution method in BHI broth on *E. faecalis* were 6.25 mg/mL and 12.50 mg/mL, respectively.

### 3.3. Intratubular Decontamination Analysis (CLSM)

**Figure 2:**
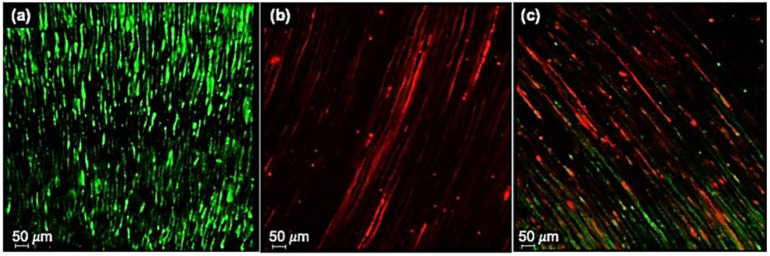
Representative images by CLSM of the untreated positive control and the two treatment groups. (**a**) Positive control: green fluorescence is evident, confirming extensive contamination with live bacteria. (**b**) NaOCl + citric acid 10% group: red fluorescence spots are predominant, indicating dead bacteria. (**c**) NaOCl + citric acid 1% group: some dots of green fluorescence can be identified, showing residual live bacteria, different from (**b**). Magnification ×40. Bars: 50.0 µm.

Representative pictures of the non-treated (positive control) and treated infected dentin (two experimental groups) are illustrated in Figure 2.

While evaluating the percentage of viable bacteria in the different groups, the D’Agostino and Pearson and Shapiro–Wilk normality test demonstrated that the distribution of values in all samples was not Gaussian (*p* < 0.05 for all groups). The 5% NaOCl + 10% CA group (Figure 2b) specimen showed the smallest number of viable bacteria; a few spots were visible. The specimen from the 5% NaOCl + 1% CA group (Figure 2c) showed a few more green dots than the other experimental specimens (Figure 2b), demonstrating greater residual viability of bacteria in the dentinal tubules. The positive control (Figure 2a) showed a more significant proportion of viable bacteria than the samples of the other two groups. Table 2 summarizes descriptive statistics and comparisons between groups. After irrigation, the percentage of viable bacteria in group I was significantly lower than in group II. In the first group, viable bacteria were nearly four times lower than in the second group (9.70% vs. 35.2%). All experimental groups had lower bacterial levels than the positive control group. No bacteria were detected in the negative control. 

Two subgroup analyses were carried out using Wilcoxon matched-pairs signed-rank test to juxtapose the proportion of viable residual bacteria in the cervical versus apical portions and the superficial versus deep layer of the dentinal tubules concerning the main root canal. In the cervical and apical parts, both treatments promoted a reduction in viability without significant differences (*p* > 0.05), exhibiting significantly lower bacterial counts than the positive control group (*p* < 0.001). The different root thirds found no significant intragroup difference in bacterial viability. When comparing bacterial viability at different depths, differences were illustrated among the experimental groups at the superficial level (*p* = 0.005). At the same time, in the deep layer, NaOCl + 10% citric acid did not produce less viable bacteria than NaOCl + 1% citric acid (*p* = 0.220). Both experimental groups showed significantly lower bacterial counts than the positive control group at shallow and deep levels. The percentage of viable residual bacteria was higher in the deeper layers than in the superficial layers in GI and GII, but without significant differences (*p* > 0.05).

## 4. Discussion

The current study assessed the cytotoxic and antimicrobial effects through a macrodilution test and intratubular contamination of different CA concentrations. To date, the Regulation (EU) 2017/745 of the European Parliament regarding medical devices [33], repealing Council Directives 90/385/EEC and 93/42/EEC, is fully applicable. According to that regulation, many medical devices, including irrigating solutions, need more self-monitoring and conformity assessment because they do not have the necessary safety and compliance requirements for patients’ safety, including non-toxicity. CA has been used for a long time in endodontics [8,34] and has been proposed instead of EDTA in combination with NaOCl as an alternative to removing the smear layer [35]. However, the cytotoxicity of CA and its safe clinical use have been little studied in recent years. Unfortunately, few published studies appear in endodontic literature containing conflicting results concerning its toxicity or not dating back over 10 years (https://pubmed.ncbi.nlm.nih.gov/?term=cytotoxicity+of+citric+acid+as+endodontic+irrigant+solution&filter=simsearch3.fft&size=20, accessed on 7 March 2022). However, some authors have shown that 10% citric acid solution did not hamper cell growth and viability, proving not cytotoxic in vitro [36,37]. In those researches, cytotoxicity was investigated on 10% CA in diluted forms compatible with cell survival (neutral pH) but not helpful in cleaning root canals, compared to the undiluted solutions (acidic pH), like presently used in our study, then more similar to the clinical practice. By an established method for in vitro cytotoxicity [25], our findings showed severe toxicity of 10% CA and diluted 1/10 corresponding to a concentration of 1%, in line with other authors [22,38]. This toxicity can be credited to the acidic nature of CA (pH 1.45 unpublished laboratory data), contributing in a significant way to killing cells also at 1/10 dilution (pH 2.1, unpublished laboratory data) [22]. With an increased dilution of the test agent at 1/100 dilution (0.1%), its toxic effect, as shown in Figure 1, vanished similarly to the negative control. Navarro-Escobar et al. [39] pointed out that the highest percentage of cell viability was obtained with 0.1% (pH 7.40) if compared to 0.5% (pH 3.54) dilution. In line with the investigation mentioned above [39], the present study showed a similar pH value (pH 7.2, unpublished laboratory data), confirming that reducing the pH near to neutrality improves cell survival of citric acid, according to a recent investigation [26]. Unfortunately, CA buffered solution, while satisfying safety and compliance requirements for patients’ safety [25,33], negatively impacts its smear layer removal capability and is therefore not usable for this purpose. It has been reported that to declare a medical device compliant, as well as not be cytotoxic, it should not be irritating for skin and oral mucosa [26].

Furthermore, it is known that tests for irritation and skin sensitization (http://nhiso.com/wp-content/uploads/2018/05/ISO-10993-10-2010.pdf, accessed on 20 April 2022) cannot be performed if the pH of the test sample is ≤2.0 or ≥11.5 because it is not desirable for animal tests to safeguard their welfare (http://nhiso.com/wp-content/uploads/2018/05/ISO-10993-2-2006.pdf, accessed on 20 April 2022). This test was not used in the present investigation due to the acidic nature of 10% and 1% solutions. As stated by some researchers [40], cultured cells are generally more sensitive to the toxic effects of materials than in vivo tissues. In in vivo conditions, substances are diluted with body fluids, and their concentration decreases by being carried away from the vascular and lymphatic systems, blood, and phagocytes. In equal concentrations, the cytotoxicity of materials diminishes along with time in the clinical setting in contrast to an in vitro observation [36]. Hence, the current findings might not be directly correlated to the clinical situation [41].

The antimicrobial activity of CA was assessed by the macrodilution method, exposing different concentrations of the irrigant to a standardized bacterial suspension by direct contact and subsequent sowing in agar plates for colony count (CFU/mL) concerning the positive control. Moreover, bacterial viability was investigated in once contaminated dentinal tubules using the CLSM imaging technique. To date, to our limited knowledge, this study is the first that has assessed the bactericidal action of citric acid inside dentinal tubules and its optimal antimicrobial concentration against *E. faecalis* determined by minimal inhibitory concentration (MIC). Instead, its minimal bactericidal concentration (MBC) had been determined more than 10 years earlier only by Arias-Moliz et al. [5].

**Figure 3:**
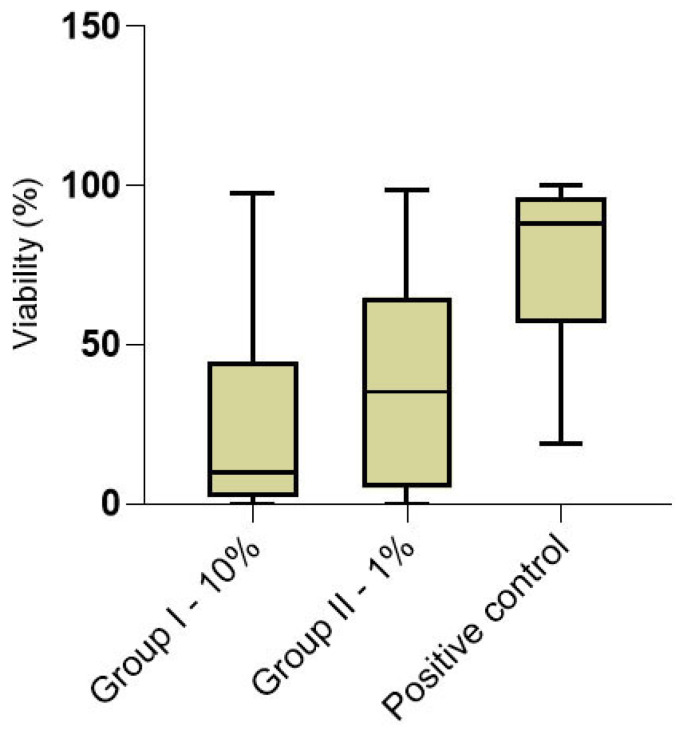
The box graph shows the percentage of viable bacterial cells in the two groups treated with irrigating solutions and the control group (right). The horizontal line inside the box is the median value.

Our results furthermore demonstrated that the concentrations of citric acid tested were effective in reducing bacterial viability within the dentinal tubules in contrast to the positive control (*p* > 0.05), with the lowest values associated with CA 10% (Figure 3), in accordance with the results of previous authors [42,43], where 10% citric acid was more effective on anaerobic bacteria and on the facultative strain *E. faecalis* than 5.25% sodium hypochlorite. Hence, the null hypothesis tested was rejected. Furthermore, Kaushik et al. [42] observed that citric acid eliminates the smear layer and has an antimicrobial action even if inferior to NaOCl. Therefore, these two chemicals were used in sequence to improve decontamination and clean the dentinal tubules.

When the median values of groups I and II were compared, the proportion of remnant viable bacteria in the first group was found to be three times lower than in NaOCl + 1% citric acid. This fact can be accredited to the lower pH value of 10% citric acid and the antimicrobial action of sodium hypochlorite [18]. Despite the excellent properties that make it the most widely used irrigant in endodontic treatment, NaOCl does not exert its effects on the inorganic constituents of the smear layer and requires the additional action of a chelator for debridement. In their studies, some authors reported that NaOCl antibacterial activity, regardless of its concentration, was lesser with the presence of the smear layer contrary to its absence [44,45]. This drawback is related to the incapacity of the solution to eradicate the smear layer and the reactivity with the organic constituents of the smear layer, which might expedite the depletion of the free available chlorine, consequently inactivating the solution. In a previous investigation [31], irrespective of the class of chelator used (CA or EDTA) as final rinse combined with NaOCl, the authors demonstrated that the antimicrobial efficacy of irrigating solutions improved and revealed a most significant percentage of dead cells in the root canal system (over 96%), possibly due to enhanced elimination of the smear layer and their deeper penetration into dentinal tubules. In the present study, a CA concentration of less than 10% was sufficient to inhibit the growth of *E. faecalis* and cause its death (0.625 mg/mL and 1.25 mg/mL, respectively). Previously, Arias-Moliz et al. [5], evaluating the MBC of CA at different concentrations and 17% EDTA, demonstrated that the latter did not exert antibacterial activity against *E. faecalis*, unlike CA, which at a concentration of 10% proved to be lethal for these bacteria, according to our findings. The antibacterial efficacy of this acidic solution may derive from the release of hydrogen ions, which would inhibit bacterial metabolism [5].

**Figure 4:**
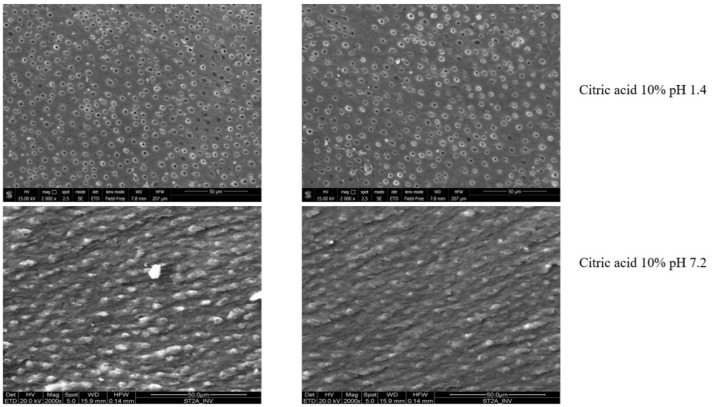
Representative scanning electron microscopy images of root canal walls after final rinse with 10% citric acid at acidic and neutral pH for two minutes. Top: smear layer is removed with dentinal tubules visible using the acidic solution. Bottom: a uniform coating of smear layer covering dentinal tubules was observed on the sample’s surface treated with the solution at neutral pH. Original magnification, 2000×.

The choice of 10% citric acid is related to its efficiency in removing the smear layer [34,37,46] and antimicrobial action on oral biofilms and anaerobic bacteria due to its acidic pH compared to EDTA [8,18,19,20]. Because of its cytotoxic potential related to its acid pH, buffered solutions at neutral pH, compliant with current regulations [32], have been investigated [13,14]. As previously highlighted [47], the pH of the citric acid solution is a crucial factor in cleaning the root canal walls than concentration. Haznedaroğlu’s study [14] reported that at pH 6, only 20% of samples showed canal wall free of smear layer if compared to its acidic formulation (90% of samples), according to our preliminary laboratory study (Figure 4).

A strain of *E. faecalis* was used to evaluate its antimicrobial efficacy at different concentrations. Although endodontic infections are polymicrobial, this bacterial strain was chosen as a tracer indicator due to the following reasons: it is the most frequent strain in persistent infections and retreatments [29,32]; it is extensively utilized to test the effectiveness of disinfecting agents in endodontics; and, lastly, it can invade and colonize dentinal tubules in depth, thus tolerating centrifugation [29]. Moreover, most bacterial species involved in root canal infections are strictly anaerobes [29] that would not survive the centrifugation process owing to airflow and forces [29].

According to previous studies, human teeth were used [27,30] to simulate clinical conditions better. Because in the apical third, mainly in human teeth, the dentin surface is sclerotic and has a lower number of tubules, with less possible contamination and penetration of the irrigating solutions, the apical portion of the samples was eliminated [26,30]. Another modification made in the present study was the use of a composite layer on the apical part of each sample to mimic an ex vivo closed-end model [30] instead of exposing the dentinal walls to direct contact with irrigants (ex vivo open-end model) as previously recommended [29], which does not reproduce the dynamics that occur within the root canal system in the course of an in vivo endodontic treatment [30].

One of the shortcomings of this study was the type of teeth used. In this study, single-rooted teeth with straight canals were used, which are relatively easy to disinfect. However, the same antibacterial effect of CA should be observed in complex root canal systems of teeth such as mandibular molars, and then it should be evaluated. Moreover, the present study evaluated only the cytotoxicity of two different concentrations of CA. Therefore, further studies evaluating CA’s genotoxicity should be performed. On the basis of the present study, citric acid 10% could be considered a valid alternative in the final irrigation of root canals. Although the CA-based irrigating solutions used in this investigation were not able to eradicate 100% of the bacteria from the dentinal tubules (90.3% in Group I and 64.8% in Group II, respectively), in clinical practice, the use of irrigants with a higher antimicrobial efficacy and no cytotoxicity that reduce the bacterial load to levels compatible with the repair of peri-radicular tissues would be preferable. Unfortunately, these promising antibacterial properties herein observed were negatively balanced by critical issues such as their toxicity, related to acidic pH, also found at low concentrations (1%). To overcome this drawback, a lower concentration than 1% citric acid (0.1%, 0.5%) previously has been suggested by some authors, preventing cell death by adjusting the chelating agent pH at a neutral value [23,34]. Recently, however, a study [25] has shown that a diluted concentration of less than 1% citric acid does not show antibiofilm and antibacterial activity, nullifying an important property required for a solution intended for effective clinical use. Therefore, further studies should be performed to evaluate various concentrations of CA for its cytotoxicity and antibacterial properties for effective and safe root canal cleaning.

## 5. Conclusions

Under the experimental conditions of this investigation, citric acid 10% showed greater antimicrobial efficacy in dentinal tubules than citric acid 1%. However, despite these valuable properties, the cytotoxic effects of citric acid at various concentrations on cell cultures must also be considered when an irrigant solution is to be used during endodontic treatment, ensuring its safe clinical use. Therefore, irrigants with similar characteristics but compliant with current regulations should be developed in the future.

## Figures and Tables

**Table 1:** Grades of cytotoxicity scored according to ISO 10993-5 2009 standard [25].

Grade	Reactivity	Reactivity Description
0	None	Discrete intracytoplasmic granules; no cell lysis
1	Slight	Not more than 20% of the cells are round, loosely attached and without intracytoplasmic granules; occasional lysed cells are present
2	Mild	Not more than 50% of the cells are round and devoid of intracytoplasmic granules; no extensive cell lysis and empty areas between cells
3	Moderate	Not more than 70% of the cell layers contain rounded cells or are lysed
4	Severe	Nearly complete destruction of the cell layers

**Table 2:** Percentage of viable bacteria in different groups.

	Group I	N	Group II	N	Positive Control	N	GI vs. GII *p-Value*	GI vs. C + *p-Value*	GII vs. C + *p-Value*
Overall	9.70 (2.18–44.60)	10	35.2 (4.87–64.90)	10	88.17 (56.81–95.13)	4	0.019	<0.001	<0.001
Cervical portion	12.86 (2.03–61.92)	10	34.80 (9.42–71.50)	10	88.17 (55.94–96.13)	4	0.051	<0.001	<0.001
Apical portion	8.0 (2.92–44.6)	10	38.46 (1.95–62.57)	10	88.29 (56.81–94.66)	4	0.213	<0.001	<0.001
C vs. A *p*-value	0.527	10	0.644	10	0.782	4			
Superficial layer	9.10 (2.03–38.98)	10	39.40 (4.95–64.53)	10	89.58 (71.38–95.06)	4	0.048	<0.001	<0.001
Deep layer	11.4 (2.22–47.67)	10	34.44 (4.87–69.11)	10	85.39 (50.79–96.13)	4	0.220	<0.001	0.002
S vs. D *p*-value	0.237	10	0.762	10	0.561	4			

Data expressed as median and 95% confidence intervals. Group I (GI): NaOCl + citric acid 10%, group II (GII): NaOCl + citric acid 1%, N: number of samples per group, C: cervical, A: apical, S: superficial, D: deep, C+: positive control group.

## Data Availability

The data presented in this study are available on request from the corresponding author.
